# Mr. Merriman, in Answer to Dr. Rowley

**Published:** 1806-03

**Authors:** S. Merriman

**Affiliations:** Curzon Street


### *To the Editors of the Medical and Physical Journal.*

GENTLEMEN,

IN the second Edition of Dr. Rowley's Pamphlet against the Cow-pock, page 90, is mentioned the case of Mr. Johnson's son, No. 16, South Moulton Street, who is therein stated to have had the small-pox, after having passed through the vaccine disease under my care. To most persons, the mere circumstance of the publication of this case in Dr. Rowley's Book, and the ungrammatical and perplexed language in which it is related, will be a sufficient proof that the statement is inaccurate. I have already alluded to this case in "*Observations on some late attempts to depreciate the value and efficacy of vaccine inoculation,*" lately published, and have there said enough to satisfy any person, at all conversant with the small-pox, that the eruption which appeared on Mr. Johnson's child, was void of every characteristic symptom of that disease, as *it had completely dried away before the expiration of three days.*

Dr. Rowley asserts, on the authority of Mr. Johnson, that when this child had been vaccinated, I offered to forfeit one hundred pounds if he should ever afterwards have the small-pox. Though I do not recollect making such a declaration, yet I belive it possible that this part of Dr. Rowley's story may be true; and therefore, that I may not be worse than my word, if he can prove to the satisfaction of twelve impartial and competent judges, that Mr. Johnson's child, whom I vaccinated, did really pass through the *genuine small-pox*, I hereby again promise to forfeit the above sum of one hundred pounds, not to Mr. Johnson, who would be above taking the money; but to the persons, styling themselves the Antivaccinarian Society, the funds of which, I understand, are at so low an ebb, that this money would be a very desirable acquisition to them.

Some

Some other cases in which I have been concerned, are much misrepresented in Dr. Rowley's pamphlet.

Cases 317, 318, 319, three children of Mr. Parnell, of No. 7, Shepherd's Market, May Fair, are stated to have had the small-pox after having been vaccinated either by Dr. Merriman or me. It is true, that these children were attempted to be vaccinated by me, but it was with dry matter, kept a day or two upon the lancets, the children not having been brought to be inoculated on the day appointed. At the time of inoculation, I expressed a fear that it would be unsuccessful; it was completely so: no more effect was produced, than if the cuticle had been gently abraded by a clean lancet. The friends of the children were made perfectly sensible, that they were not secured from the casual infection of the small-pox, and were enjoined to send them to be inoculated again, but neglected it. This is the simple truth of a story, thus artfully trumped up to deceive and mislead the public. O, shame! where is thy blush?

Another false report in Dr. Rowley's pamphlet is respecting an eruption on Mr. Turtle's child, No. 199, page 64. Dr. R. calls the disease "the cow-pock mange treated unsuccessfully as the itch." The child, he says, "is now two years old, Aug. 29, 1805, under my care." That the child had contracted the *common itch*, by contagion, fourteen months after vaccination, I can assert on the authority of a physician of more than fifty years experience. The treatment which she then underwent was not unsuccessful, as the child was cured by it. She has since been occasionally subject to the prurigo, as described by Dr. Willan, Order I. Class iii. sometimes to a very considerable degree; for which, many more probable causes may be assigned than the cow-pock, particularly the eating of strong cheese, and drinking porter, which articles of diet I lately saw her enjoying, with much apparent satisfaction. Lastly, the child could not be said to be under Dr. Rowley's care: the medicines he ordered, did the child no good, but put her to *most excruciating pain*; they were, therefore, soon discontinued; yet, when the colder weather set in, the prurigo, as is usual, spontaneously disappeared.

Having the pen in my hand, I cannot forbear congratulating the public, and in particular the friends of the Jennerian discovery, on the victory obtained by vaccination, in the very head quarters of the enemy. The Governors of the Marybone Infirmary have, I am informed, given

*peremptory*

*peremptory orders,* in utter contempt of Dr. Rowley's pamphlet, that every child belonging to that extensive charity shall be vaccinated. When it is recollected, how many years Dr. Rowley has been physician to that well conducted establishment, how long he has there reigned lord paramount, this must be considered a triumph of the cause of Truth and Vaccination, as important as it is decisive.

I am, &c.

*Curzon Street, Feb.* 6, 1806. S. MERRIMAN, Jun.

